# Is postpartum depression a homogenous disorder: time of onset, severity, symptoms and hopelessness in relation to the course of depression

**DOI:** 10.1186/s12884-014-0402-2

**Published:** 2014-12-10

**Authors:** Pirjo Kettunen, Eeva Koistinen, Jukka Hintikka

**Affiliations:** Department of General Hospital Psychiatry, North Karelia Central Hospital, Joensuu, Finland; Department of Obstetrics and Gynecology, North Karelia Central Hospital, Joensuu, Finland; School of Medicine, University of Tampere, Lahti, Finland; Department of Psychiatry, Paijat-Hame Central Hospital, Lahti, Finland

**Keywords:** Postpartum depression, Pregnancy, Delivery, Depression, Symptoms, Hopelessness, General population

## Abstract

**Background:**

Postpartum depression (PPD) is a common illness, but due to the underlying processes and the diversity of symptoms, some variability is exhibited. The risk of postpartum depression is great if the mother has previously suffered from depression, but there is some evidence that a certain subgroup of women only experience depression during the postpartum period.

**Methods:**

The study group consisted of 104 mothers with postpartum major depression and a control group of 104 postpartum mothers without depression. The Structured Clinical Interview for DSM-IV Axis I Disorders (SCID-I) was used for data collection. The severity of depression and other mental symptoms were assessed using several validated rating scales.

**Results:**

A history of past depression (82%), including depression during pregnancy (42%) and during the postpartum period (53%), was very common in those with current PPD. Eighteen per cent of mothers with current PPD had previously not had any depressive episodes and four per cent had experienced depression only during the postpartum period. Therefore, pure PPD was rare. The onset of PPD was usually (84%) within six weeks of childbirth. Obsessive-compulsive symptoms, phobic anxiety, paranoid ideation, depressed mood, diminished pleasure/interest, decreased energy, and psychomotor agitation/retardation were common with all kinds of depression histories. Pure PPD was the most similar to the first depressive episode. Nevertheless, the severity of depression, the level of hopelessness, somatisation, interpersonal sensitivity, anxiety, hostility, psychoticism, sleep disturbance, and suicidal ideation were lower, appetite changed less, and concentration was better than in other recurrent depressions.

**Conclusions:**

According to this study, PPD is not a homogenous disorder. The time of onset, severity, symptoms, level of hopelessness, and the course of depression vary. Recurrent depression is common. All mothers must be screened during the sixth week postpartum at the latest. Screening alone is not effective; it is also important to give mothers information about PPD and to discuss the symptoms with them in order for them to recognise this disorder and possible new episodes in the future.

## Background

Depressive symptoms are among the most frequent psychiatric manifestations observed in women after childbirth. In their meta-analysis, O’Hara and Swain [1] found an average prevalence rate of 13% and a variation depending on the methods of assessment and time of onset after childbirth. A systematic review by Gavin et al. [2] suggested that 19.2% of women suffer from major or minor depression (7.1% for major depression alone) during the first three months postpartum, and the prevalence varies from 6.5% to 12.9% (1.0% to 5.6% for major depression) during the postpartum year. However, Halbreich and Karkun [3] found that an average prevalence of 10–15% for postpartum depression (PPD) is not representative of the actual global prevalence due to the variability of the underlying processes and the diversity of symptoms. The postpartum period is unique with respect to psychosocial adjustment and the degree of neuroendocrine alterations.

Although PPD is common, it is often missed by primary care teams [4]. Moreover, mothers often face barriers to seeking help, such as an inability to disclose their feelings or to recognise the symptoms of depression; they also lack of knowledge about postpartum depression [5].

It is not easy to differentiate PPD from other psychiatric and non-psychiatric disorders or to separate depressed mothers from healthy mothers. Patterns of appetite, especially loss of appetite [6], fatigue, and disrupted sleep related to infant care may be difficult to distinguish from the symptoms of depression. Sleep disturbances can also lead to negative consequences, such as dysphoric mood and impaired cognitive function, while persistent difficulty with concentration or cognitive tasks is indicative of a mood disorder [7]. Substance abuse and medical causes of psychiatric symptoms, such as thyroid disorders, should also be considered [8]. The “baby blues” is a transient mood disturbance that can affect as many as 70% of new mothers within ten days of delivery. It manifests as tearfulness, irritability, anxiety, and emotional liability, as well as interpersonal hypersensitivity, insomnia, and sometimes elation, but it does not impair function [9,10]. Mothers with a rapid onset of intense mood disturbance, confusion, strange or delusional beliefs, hallucinations, and disorganised behaviour have symptoms of postpartum psychosis, which is most commonly a form of bipolar disorder [11]. Other forms of bipolar I–II disorders should also be considered [12]. Co-morbidity studies suggest that PPD is associated with generalised anxiety disorders, panic disorders, post-traumatic stress disorders, and obsessive-compulsive disorders [13-16]. The incidence of personality disorders among PPD patients is greater than among non-PPD patients, and many symptoms are coloured by these disorders [17].

In different meta-analyses, the following risk factors have been found to have a moderate to strong association with PPD: depression and anxiety during pregnancy, a previous history of depression, stressful life events (including child care-related stressors), a poor marital relationship, and poor social support [1,18,19]. Other risk factors, including a low socioeconomic status, unplanned/unwanted pregnancy, obstetric factors, and difficult infant temperament are less strongly related to PPD [18,19]. The risk for depression is greater if the mother has had psychiatric illnesses before, including illnesses during pregnancy. The risk is especially high if the mother has experienced depression during previous postpartum periods or at other times [1,19-21].

There is some evidence that a subgroup of women have depression only during the postpartum period. Cooper and Murray [22] have compared primiparous women whose PPD was a recurrence of a prior non-postpartum mood disorder with a group of women for whom PPD was their first experience of affective disturbance. The former group was found to be at a greater risk for subsequent non-postpartum depression, whereas the latter group was found to be at a greater risk for subsequent PPD.

Rapid changes in hormonal levels following delivery have been suggested to predispose women to PPD. One might expect that these changes would affect the symptoms, incidence, and type of depression over the perinatal period [23-25].

Depressive symptoms are uncommon during the immediate postpartum period. As observed in a recent study, only 2.5% of women acknowledged symptoms of major depression within the first two days of delivery [26]. The low figure may be caused by the fact that not all the physiological changes have occurred yet and the stress of childcare is yet to come. Incidences of depression increase significantly during the first three months after delivery [20,27,28], and the incidence of depression may be threefold higher five weeks after childbirth [29]. Kumar and Robson [27] found that incidences of depression decreased after six months and that there was no increase at a year after delivery. Depression may last for several months [20,27,29]. The prevalence of depression does not increase during pregnancy. Indeed, depressive episodes are 3.5 times more prevalent during the postpartum period than during pregnancy [2,21].

The symptoms of PPD also vary according to many studies. In her pioneer study, Pitt [30] found that PPD is atypical, either because of the prominence of neurotic symptoms such as anxiety, irritability, somatic symptoms, fatigue, and phobias overshadowing the depression, or because some features are the opposite of those of classical depression. For example difficulties in falling or staying asleep may be common instead of early awakening [31]. Only few of patients exhibited the classical picture of depressive illness with suicidal ideas. Miller et al. [32] found much higher rates of obsessive-compulsive symptoms among those suffering from PPD than among subjects of population-based studies. Women with postpartum-onset major depression experience disturbing aggressive obsessional thoughts more frequently than women with non-postpartum major depression [33]. Postpartum anxiety symptoms, including somatic and social anxiety and comorbid depression symptoms, have been found to be fairly common: 10–50% of mothers exhibit these symptoms [34,35]. According to Bernstein et al. [36], psychomotor symptoms and impaired concentration/decision-making were prominent in PPD, while women who had depression outside the postpartum period more often reported a sad mood, suicidal ideation, and reduced interest. Prevalence of thoughts of self-harm and suicidal ideation during the postpartum period was between 5% and 14% [37,38].

The mood in a major depressive episode is often described by the person as sad, hopeless, and discouraged [10]. Hopelessness has been identified as one of the core characteristics of depression. A common denomination in depression is a pattern of negative expectations of the future [39]. It is generally supposed that childbirth induces hope and positive expectations of the future. However, many mothers with PPD express hopelessness at a time when one would expect to see joy and hope [40]. Due to this contradiction, more studies are needed on hopelessness among depressed mothers.

Although PPD is common, it is often missed by primary care teams. Moreover, mothers often face barriers to seeking help, such as an inability to disclose their feelings or to recognise the symptoms of depression. It is not easy to differentiate PPD from other psychiatric and non-psychiatric disorders or to separate depressed mothers from healthy mothers. The postpartum period is unique with respect to psychosocial adjustments and the degree of neuroendocrine alterations, and there seems to be variability due to the underlying processes and the diversity of symptoms. The course of depression also seems to be different. In the context of our study, we are interested to learn whether PPD is homogenous according to the course of depression. In addition, we wish to learn more about the time of onset of PPD, its severity, its symptoms, and the level of the hopelessness experienced. The results may have clinically important implications for the detection, screening, and further treatment of this disorder.

The study was designed to assess the course of depression, time of onset, severity of symptoms, the symptom profile, and the level of hopelessness in order to find out:

- whether mothers with PPD are different from mothers without PPD in relation to the severity of symptoms, symptom profile, and level of hopelessness;
- whether mothers with PPD have experienced more incidences of depression than mothers without PPD;
- whether recurrent depression differs from the first depressive episode;
- whether PPD with depression during pregnancy (DDP) is different from PPD without DDP;
- whether pure PPD (mothers who previously have not had episodes of depression or only PPD) is different from other types of postpartum depression.

## Methods

In Finland, a postpartum examination is offered to all mothers at six weeks after childbirth, after the puerperium [41]. Mothers were screened during this examination by primary care nurses at the antenatal clinic in Joensuu, Eastern Finland, using the Edinburg Postnatal Depression Scale (EPDS-10, range 0–30) [42]. The EPDS has been developed to assist primary care health professionals detect mothers suffering from postnatal depression. If the depressive symptoms of the mothers began later (i.e. up to six months after delivery) they contacted their antenatal clinic’s nurse, who assessed their depression using the EPDS. If the EPDS score was ≥ 10 or there was a clinical suspicion of depression, the nurse told the mother that she could be assessed by a psychiatrist (PK) at the local General Hospital Psychiatric unit of the North Karelia Central Hospital in Joensuu. This community-based hospital unit serves a socioeconomically diverse population. All mothers who wanted to attend the psychiatric unit were evaluated by a psychiatrist at six weeks to six months after delivery. Mothers with psychotic, addictive and thyroid disorders were excluded from the study.

Diagnoses of major depressive disorder in the study group (depressed mothers) and the control group (non-depressed mothers) were assessed by a psychiatrist (PK) by means of the Structured Clinical Interview for DSM-IV (*Diagnostic and Statistical Manual of Mental Disorders*, fourth edition) Axis I Disorders (SCID-I) [10,43]. DSM-IV uses the term “postpartum onset” as a specifier applicable to major depressive disorder, bipolar disorder or brief psychotic disorder occurring over the first four weeks following childbirth [10]. The diagnostic criteria for a Major Depressive Episode (MDE), as defined by the DSM-IV, include five (or more) of the following symptoms: at least two weeks of persistent depressed mood, loss of interest/pleasure, increased or decreased appetite, sleep disturbance, psychomotor agitation or retardation, decreased energy, feelings of worthlessness or guilt, poor concentration, and suicidal ideation. At least one of the symptoms must be either depressed mood or loss of interest. For this study, all nine SCID symptoms – the entry criteria (depressed mood and/or loss of interest) and seven associated symptoms – were assessed in the depressed and non-depressed group. This procedure (i.e. an adaptation of the usual SCID procedure) allows comparisons of all SCID symptoms between groups.

The groups were asked questions about the onset of depression and experiences of previous depressive episodes as a part of the semi-structured SCID interviews. Previous depressive episodes were assessed by asking whether the mothers had had persistent depression (i) for at least two weeks without connection to their pregnancy or postpartum period, (ii) during pregnancy, (iii) and/or during previous pregnancies, and (iv) whether they had had previous postpartum depression in the period up to six months after delivery. The answers were classified as yes or no. The onset of PPD was assessed by asking how soon after childbirth the depression began. This was reported as weeks after delivery. The mothers were also asked if they had given birth to a living infant or not, and how many children they had.

The severity of depression was rated using the number of MDE-positive symptoms found in the SCID interview, the self-administered 21-item Beck Depression Inventory (BDI-21, range 0–63) [44], and the 10-item EPDS. The level of hopelessness was assessed by the 20-item Beck Hopelessness Scale (BHS-20, range: 0–20) [39].

Mental symptoms were assessed by the 90-item Symptoms Checklist 90 (SCL-90) [45]. The SCL-90 measures symptom intensity on nine different subscales, including somatisation, obsessive-compulsive symptoms, interpersonal sensitivity, depression, anxiety, hostility, phobic anxiety, paranoid ideation, and psychoticism. The SCL-90 is capable of differentiating healthy and mentally ill subjects, and its discriminant validity is good in the Finnish population [46]. High intercorrelations were found between the nine original subscales. However, the checklist is not capable of distinguishing between different diagnostic groups [45,46].

The study protocol was approved by the Ethical Committee of the North Karelian Hospital District Federation of Municipalities. All participants gave their informed consent prior to data collection.

The final study group consisted of 104 mothers with a major depressive episode, aged 18–40 years. The participants expressed their willingness to participate in this study. Data collection took place from 2003 to 2013. A control group of non-depressed mothers was collected at the antenatal clinic in Joensuu. If the EPDS score was <10 in postpartum examinations, primary care nurses asked if the mothers were willing to participate in the non-depressed group of mothers and then organised psychiatric evaluation. The final control group consisted of 104 non-depressed mothers, evaluated six weeks to six months after delivery and aged 18–40 years. The control group was collected between 2008 and 2010. Mothers with psychotic, addictive and thyroid disorders were again excluded.

Data analysis was conducted with IBM SPSS (version 21). The differences between the study groups were examined with Pearson’s chi-square test and Fisher’s exact test for the categorical variables, and the independent samples t-test was used for the continuous variables. If a continuous variable was not normally distributed, the non-parametric Mann–Whitney U test was used. The statistical significance of the tests was defined as 0.05. The relationship between previous depression and current PPD was investigated using age-adjusted logistic regression.

## Results

The depressed mothers were younger than the non-depressed controls (mean 27.4 (SD 5.3) years versus 29.6 (SD 4.1), p = 0.001). The frequency of being the mother of a first living infant was similar in both groups (51.0% and 46.2%, respectively, p = 0.49). All the mothers had given birth to a living infant.

Table 1 shows the comparisons between the non-depressed and depressed groups according to the severity of symptoms and symptom profile. Unsurprisingly, the depressed group reported significantly more symptoms of depression in the SCID interview and higher EPDS and BDI scores than the non-depressed group. In addition, the BHS score was higher among depressed mothers. All nine symptoms of the SCID interview were significantly different. Moreover, they were more symptomatic on all nine subscales of the SCL-90.

**Table 1:** **Comparisons between the non-depressed and depressed groups according to the severity of depression and symptoms**

	**Non-depressed group n = 104**	**Depressed group n = 104**	
	**Mean (SD)**	**Mean (SD)**	**p-value**
Severity of depressive symptoms:			
Number of symptoms^3^	0.29 (0.72)	6.54 (1.29)	<0.001^1^
EPDS score	3.53 (3.20)	17.59 (4.08)	<0.001^1^
BDI score	4.08 (3.17)	22.22 (7.95)	<0.001^1^
BHS score	1.98 (1.91)	7.69 (4.50)	<0.001^1^
SCL-90 subscales:			
Somatisation	1.38 (0.36)	2.18 (0.71)	<0.001^1^
Obsessive-compulsive	1.46 (0.41)	2.75 (0.63)	<0.001^1^
Interpersonal sensitivity	1.23 (0.30)	2.38 (0.73)	<0.001^1^
Depression	1.43 (0.40)	3.21 (0.60)	<0.001^1^
Anxiety	1.17 (0.24)	2.35 (0.73)	<0.001^1^
Hostility	1.34 (0.36)	2.36 (0.73)	<0.001^1^
Phobic anxiety	1.08 (0.18)	1.91 (0.76)	<0.001^1^
Paranoid ideation	1.16 (0.26)	1.86 (0.67)	<0.001^1^
Psychoticism	1.06 (0.14)	1.68 (0.52)	<0.001^1^
Symptoms of major depressive episode in SCID interview:	n (%)	n (%)	p-value
Depressed mood	5 (4.8 )	93 (89.4)	<0.001^2^
Loss of interest/pleasure	4 (3.8)	86 (82.7)	<0.001^2^
Increased or decreased appetite	1 (1.0)	34 (32.7)	<0.001^2^
Sleep disturbance	1 (1.0)	68 (65.4)	<0.001^2^
Psychomotor agitation or retardation	4 (3.8)	80 (76.9)	<0.001^2^
Decreased energy	5 (4.8)	97 (93.3)	<0.001^2^
Worthlessness/feelings of guilt	5 (4.8)	90 (86.5)	<0.001^2^
Poor concentration	7 (6.7)	80 (76.9)	<0.001^2^
Suicidal ideation	0 (0.0)	26 (25.0)	<0.001^2^

^1^Mann–Whitney U test.

^2^Pearson’s chi-square test.

^3^Number of symptoms of a major depressive episode in the SCID interview

EBDS = Edinburgh Postnatal Depression Scale, BDI = Beck Depression Inventory, BHS = Beck Hopelessness Scale, SD = standard deviation.

The depressed mothers had a history of previous depression more frequently than the non-depressed mothers (85/104 (81.7%) versus 32/104 (30.8%), p < 0.001). The same applies to a history of DDP, a history of depression during previous pregnancies, a history of previous PPD, and a history of previous depression without any connection to pregnancy or the postpartum period (Table 2). Eighty-five per cent (85.2%; 23/27) of depressed mothers with a history of previous PPD and 42.9% (3/7) of non-depressed mothers had a history of both PPD and depression outside the postpartum period (p = 0.037). Four of the depressed mothers and four of the non-depressed mothers had not had any previous depression other than PPD. Twenty-two per cent (22.1%; 23/104) of the mothers had pure PPD, meaning mothers who had previously not had depression (18.3%; 19/104) or had only had PPD (3.8%; 4/104). A history of previous depression without any connection to pregnancy or the postpartum period was as common among mothers with a history of DDP as among other mothers (75.0%; 33/44 versus 60.0%; 36/60, p = 0.110).

**Table 2:** **Previous depression in the non-depressed and depressed group and age-adjusted associations for postpartum depression**

	**Non-depressed group n (%)**	**Depressed group n (%)**	**p-value**	**OR**	**95% CL**	**p-value**
Depression during pregnancy	5/104 (4.8%)	44/104 (42.3%)	<0.001^1^	14.752	2.723–79.936	0.002
Depression during previous pregnancy, mothers with previous pregnancies	3/57 (5.3%)	13/53 (24.5%)	0.004^1^	0.856	0.146–5.023	0.863
Previous postpartum depression, mothers with previous childbirth	7/55 (12.7%)	27/51 (52.9%)	<0.001^1^	6.124	1.831–20.478	0.003
Previous depression without connection to pregnancy or delivery	26/104 (25%)	69/104 (66.3%)	<0.001^1^	6.712	2.183–20.641	0.001

^1^Pearson’s chi-square test.

OR = odds ratio, CL = confidence limits.

The age-adjusted risk of PPD associated significantly with DDP, previous PPD, and a history of depression without any connection to pregnancy or the postpartum period (Table 2).

Forty-six per cent (46.2%) of the depressed mothers were depressed within 1.5 weeks, 74.0% within 4 weeks, 83.7% within 6 weeks and 98.1% within three months of childbirth. All the PPD diagnoses were reached within 22 weeks of childbirth. Mothers with a history of DDP were more often depressed within 1.5 weeks of childbirth than other mothers. There were no differences between these groups later. No differences were found in the time of occurrence between the first and recurrent episodes of depression or between pure and other types of postpartum depression (Table 3).

**Table 3:** **Presence of depression from childbirth according to previous depression**

	**History of previous depressive episode**			**History of depression during pregnancy**			**Pure postpartum depression**			**All depressed mothers**
	**No (n = 19)**	**Yes (n = 85)**		**No (n = 60 )**	**Yes (n = 44 )**		**No (n = 81)**	**Yes (n = 23)**		**N = 104**
	**n (%)**	**n (%)**	**p-value**	**n (%)**	**n (%)**	**p-value**	**n (%)**	**n (%)**	**p-value**	**n (%)**
0–1.5 weeks	9 (47.4)	39 (45.9)	0.906^1^	22 (36.7)	26 (59.1)	0.023^1^	37 (45.7)	11 (47.8)	0.855^1^	48 (46.2)
2–4 weeks	7 (36.8)	22 (25.9)	0.335^1^	18 (30.0)	11 (25.0)	0.574^1^	22 (27.2)	7 (30.4)	0.757^1^	29 (27.9)
4.5–6 weeks	0 ( 0.0)	10 (11.8)	0.202^2^	8 (13.3)	2 (4.5)	0.185^2^	9 (11.1)	1 (4.3)	0.452^2^	10 (9.6)
6.5–13 weeks	2 (10.5)	13 (15.3)	0.733^2^	10 (16.7)	5 (11.4)	0.447^1^	12 (14.8)	3 (13.0)	1.000^2^	15 (14.4)
13.5–22 weeks	1 (5.3)	1 (1.2)	0.333^2^	2 (3.3)	0 (0.0)	0.507^2^	1 (1.2)	1 (4.3)	0.395^2^	2 (1.9)

^1^Pearson’s chi-square test.

^2^Fisher’s exact test.

Table 4 shows the severity of depressive symptoms according to previous episodes of depression. The mean number of MDE symptoms, the BDI score and the BHS score were higher in a recurrent depressive episode than in the first episode, and similarly higher among those who had a history of DDP than among those without such a history. Likewise, the scores were lower in pure PPD than in other types of depression. The EPDS score was statistically significantly lower for the first episode and for pure PPD.

**Table 4:** **Severity of depressive symptoms according to previous depression**

	**History of previous depressive episode**			**History of depression during pregnancy**			**Pure postpartum depression**		
	**No (n = 19)**	**Yes (n = 85)**		**No (n = 60)**	**Yes (n = 44)**		**No (n = 81)**	**Yes (n = 23)**	
	**Mean (SD)**	**Mean (SD)**	**p-value** ^**2**^	**Mean (SD)**	**Mean (SD)**	**p-value** ^**2**^	**Mean (SD)**	**Mean (SD)**	**p-value** ^**2**^
Number of symptoms^1^	5.58 (0.61)	6.75 (1.31)	<0.001	6.28 (1.18)	6.89 (1.37)	0.024	6.77 (1.32)	5.74 (0.81)	0.001
EPDS score	15.32 (4.22)	18.09 (3.90)	0.012	17.02 (3.99)	18.36 (4.12)	0.137	18.10 (3.98)	15.78 (4.00)	0.028
BDI score	17.03 (6.27)	23.38 (7.85)	0.001	19.97 (6.98)	25.29 (8.52)	0.001	23.35 (7.84)	18.24 (7.18)	0.003
BHS score	5.52 (3.47)	8.17 (4.58)	0.016	6.42 (4.13)	9.42 (4.45)	0.001	8.32 (4.56)	5.45 (3.54)	0.006

^1^Number of symptoms in SCID interview.

^2^P-values are for Mann–Whitney U test.

EPDS = Edinburgh Postnatal Depression Scale, BDI = Beck Depression Inventory, BHS = Beck Hopelessness Scale, SD = standard deviation.

There is a great variety in the SCL-90 scores as regards to previous episodes of depression (Table 5). Of the SCL-90 subscale scores, somatisation, interpersonal sensitivity, hostility, and psychoticism were higher in a recurrent depressive episode than in the first episode, among those who had a history of DDP than among those without such a history, and among those who had had other types of depression than pure PPD. The SCL-90 depression and anxiety scores were higher among those with a history of DDP than among those without such a history and those who had had other types of depression than pure PPD. No differences were found in obsessive-compulsive symptoms, phobic anxiety or paranoid ideation.

**Table 5:** **Symptom Checklist-90 scores according to previous depression**

	**History of previous depressive episode**			**History of depression during pregnancy**			**Pure postpartum depression**		
	**No (n = 19)**	**Yes (n = 85)**		**No (n = 60)**	**Yes (n = 44)**		**No (n = 81)**	**Yes (n = 23)**	
**SCL-90 subscales**	**Mean (SD)**	**Mean (SD)**	**p-value** ^**1**^	**Mean (SD)**	**Mean (SD)**	**p-value** ^**1**^	**Mean (SD)**	**Mean (SD)**	**p-value** ^**1**^
Somatisation	1.88 (0.54)	2.25 (0.73)	0.033	2.06 (0.65)	2.35 (0.77)	0.042	2.27 (0.74)	1.88 (0.52)	0.020
Obsessive-compulsive symptoms	2.63 (0.60)	2.78 (0.63)	0.363	2.65 (0.65)	2.89 (0.56)	0.099	2.79 (0.63)	2.60 (0.60)	0.202
Interpersonal sensitivity	1.98 (0.59)	2.47 (0.73)	0.008	2.18 (0.64)	2.65 (0.78)	0.002	2.49 (0.73)	1.97 (0.57)	0.002
Depression	2.97 (0.64)	3.26 (0.59)	0.074	3.09 (0.60)	3.38 (0.57)	0.025	3.27 (0.59)	3.00 (0.61)	0.043
Anxiety	2.13 (0.57)	2.40 (0.75)	0.159	2.21 (0.69)	2.55 (0.73)	0.012	2.43 (0.75)	2.08 (0.57)	0.042
Hostility	2.03 (0.59)	2.43 (0.74)	0.034	2.22 (0.69)	2.54 (0.75)	0.028	2.44 (0.74)	2.05 (0.59)	0.023
Phobic anxiety	1.80 (0.62)	1.94 (0.79)	0.710	1.80 (0.70)	2.07 (0.84)	0.080	1.96 (0.80)	1.72 (0.59)	0.276
Paranoid ideation	1.61 (0.60)	1.91 (0.68)	0.071	1.77 (0.65)	1.98 (0.69)	0.096	1.92 (0.69)	1.65 (0.58)	0.084
Psychoticism	1.44 (0.37)	1.73 (0.54)	0.023	1.60 (0.50)	1.79 (0.54)	0.037	1.74 (0.54)	1.45 (0.36)	0.015

^1^P-values are for the Mann–Whitney U test.

SCL-90 = 90-item Symptom Checklist, SD = standard deviation.

The prevalence of the symptoms of a major depressive episode classified according to previous depression is presented in Table 6. Increased or decreased appetite, sleep disturbance, and suicidal ideation as symptoms of depression were more common in a recurrent depressive episode than in the first episode, and likewise more common in other types of depression than in pure PPD. Worthlessness/feelings of guilt were more common among those who had a history of DDP than among those without such a history. Finally, poor concentration was significantly more common among other types of depression than pure PPD. No differences were found in depressed mood, loss of interest/pleasure, psychomotor agitation/retardation, or decreased energy.

**Table 6:** **Prevalence of symptoms of a major depressive episode according to previous history of depression**

**Symptoms of a major depressive episode in the SCID interview**	**History of previous depressive episode**			**History of depression during pregnancy**			**Pure postpartum depression**		
	**No (n = 19)**	**Yes (n = 85)**		**No (n = 60)**	**Yes (n = 44)**		**No (n = 81)**	**Yes (n = 23)**	
	**n (%)**	**n (%)**	**p-value**	**n (%)**	**n (%)**	**p-value**	**n (%)**	**n (%)**	**p-value**
Depressed mood	16 (84.2)	77 (90.6)	0.418^1^	54 (90.0)	39 (88.6)	1.000^2^	73 (90.1)	20 (87.0)	0.704^1^
Loss of interest/pleasure	15 (78.9)	71 (83.5)	0.738^1^	50 (83.3)	36 (81.8)	0.840^1^	67 (82.7)	19 (82.6)	1.000^1^
Increased or decreased appetite	2 (10.5)	32 (37.6)	0.023^2^	16 (26.7)	18 (40.9)	0.126^1^	31 (38.3)	3 (13.0)	0.023^2^
Sleep disturbance	6 (31.6)	62 (72.9)	0.001^2^	35 (58.3)	33 (75.0)	0.078^1^	58 (71.6)	10 (43.5)	0.012^2^
Psychomotor agitation/retardation	16 (84.2)	64 (75.3)	0.552^1^	32 (72.7)	32 (72.7)	0.384^1^	62 (76.5)	18 (78.3)	0.863^2^
Decreased energy	18 (94.7)	79 (92.9)	1.000^1^	54 (90.0)	43 (97.7)	0.234^2^	75 (92.6)	22 (95.7)	1.000^1^
Worthlessness/feelings of guilt	14 (73.7)	76 (89.4)	0.128^1^	48 (80.0)	42 (95.5)	0.023^1^	73 (90.1)	17 (73.9)	0.077^1^
Poor concentration	12 (63.7)	68 (80.0)	0.136^1^	43 (71.7)	37 (84.1)	0.137^1^	66 (81.5)	14 (60.9)	0.038^2^
Suicidal ideation	1 (5.3)	25 (29.4)	0.038^1^	14 (23.3)	12 (27.3)	0.647^1^	24 (29.6)	2 (8.7)	0.041^2^

^1^Fisher’s exact test.

^2^Pearson’s chi-square test.

## Discussion

According to this study, PPD is not a homogenous disorder. It is usually connected with a history of previous depression, but it is also possible that a subgroup of women only suffer from depression during the postpartum period. There are many variations in time of onset, severity, symptoms, and level of hopelessness. This may be due to biological vulnerability or because of other childbirth-specific features that may predispose women to PPD and overshadow the postpartum state.

The present study, like numerous other studies [1,18-21], shows that a previous history of depression including DDP and earlier PPD, is common with PPD. A previous history of PPD is usually connected with episodes of depression outside the postpartum period. The incidence of pure PPD – where mothers had experienced no previous depression or only previous PPD – was fairly small (22.1%), and the episode was often the first one (18.3%).The question of whether the first episode denotes the beginning of a recurrent depressive disorder should be addressed in a follow-up study.

This study also shows that the BDI, EPDS and BHS scores were significantly different between the mothers suffering from major depression and the non-depressed postpartum mothers. The capacity of these scales to differentiate depressed and non-depressed mothers is good. All nine SCID symptoms and all SCL-90 symptoms were different between the mothers suffering from major depression and the non-depressed postpartum mothers. The symptom profile was wide, as shown by numerous studies [6,30-38]. Increased or decreased appetite, sleep disturbances, and decreased energy, each of which may be difficult to differentiate from healthy women because of postpartum status, other medical reasons and child care stress were also different [6-8]. Many of the symptoms are similar to those of generalised anxiety disorders, panic disorders, post-traumatic stress disorders, obsessive-compulsive disorders, and personality disorders, and co-morbidity may be high between these disorders and PPD [10,13-17].

The baby blues is common during the first ten days after delivery [9,10], and 46.2% of the mothers in the present study stated that the onset of depression occurred within this period. Pawar et al. [26] found that only 2.5% of women acknowledged major depression symptoms within the first two days following delivery. In our study, depression during the baby blues period was more common among those who had had DDP than among those who had had no DDP. It can be supposed that in some mothers, depression continues after pregnancy into the baby blues period. It must be noted, however, that the method used in this study was retrospective self-report and the period assessed was longer. There may have been a recall bias from some mothers who may have been unable to differentiate between the symptoms of the baby blues and the beginning of a major depressive episode. According to the DSM-IV, postpartum depression occurs within four weeks of delivery. Seventy-four per cent of our depressed mothers had PPD within that period. The puerperium period is six weeks, and eighty-four per cent of depressed mothers had PPD within that period. Moreover, nearly all (98%) mothers experienced a new episode of depression three months of childbirth and all of them within 22 weeks, respectively. Our results are in line with previous studies [20,27-29].

A new finding is that there were differences in the severity of depressive symptoms according to previous depression. The level of hopelessness was greater and the number of SCID symptoms and the BDI score were lower in the first episode of depression, in depression without DDP and in pure PPD than in other types of depressions. The EPDS score, the specific measure to PPD, was significantly lower in the first depression and in pure PPD.

Adding to the current literature, it can be stated that there was a great variety in symptoms with PPD according to previous episodes of depression. Anxiety and obsessive-compulsive symptoms are common with PPD according to earlier studies [30,32-35]. Obsessive-compulsive symptoms, phobic anxiety, and paranoid ideation were common with all kinds of depression histories. The symptoms of pure PPD were very similar compared to the first depression episode and to depression without a history of DDP. Somatisation, interpersonal sensitivity, hostility, and psychoticism were lighter in the first episode of depression than in recurrent episodes of depression and among those without a history of DDP compared to those who had. The same applies to those having pure PPD as opposed to those having other types of depression. In this study, anxiety was less severe among those without a history of DDP and in pure PPD.

An additional new finding is that MDE symptoms vary according to previous episodes of depression. According to previous studies, psychomotor symptoms, decreased energy, changes in appetite, sleep disturbances, poor concentration, and mild suicidal ideation are typical of PPD [6,30,31,36]. In this study, symptoms such as depressed mood, loss of interest/pleasure, psychomotor agitation/retardation, and decreased energy were common with all kinds of histories of depression. The diagnostic criteria for MDE include depressed mood or loss of interest [10], and these criteria may be of a greater importance because of the definition of MDE. Furthermore, changes in appetite, sleep disturbances, and suicidal ideations were less prevalent in the first episode of depression than in recurrent episodes and in pure PPD than in other types of depression. Poor concentration was only significantly less prevalent in pure PPD. If mothers had experienced DDP, their feelings of worthlessness were stronger, which may be a result of negative experiences during pregnancy.

One of the limitations of the present study was the use of a retrospective self-report about depression episodes and their severity, duration, and time of onset. Women may have over- or underestimated their responses in self-report questionnaires according to their beliefs and perceptions. Furthermore, the sample was a convenience sample. We could only evaluate mothers who accepted an invitation by the primary care nurses to attend a general hospital unit because of depressive symptoms. Nevertheless, the depression group and the healthy control group represent the same population. The diagnostic interview constitutes a critical strength of the study. The diagnoses of major depressive disorders in the study group and the control group were assessed by a psychiatrist by means of the Structured Clinical Interview for DSM-IV Axis I Disorders. One limitation is the fact that the psychiatrist (PK) conducting the clinical interviews was not blind to the mothers’ depression status.

## Conclusions

According to the present study, PPD is not a homogenous disorder, and it is usually connected to a previous history of depression. The time of onset, severity, and level of hopelessness vary according to the course of the depression, and symptom profile is wide. Obsessive-compulsive symptoms, phobic anxiety, paranoid ideation, depressed mood, diminished pleasure/interest, decreased energy, and psychomotor agitation/retardation were common with all kinds of depression histories. Pure PPD was the most similar compared to the first depressive episode. Nevertheless, the severity of depression, level of hopelessness, somatisation, interpersonal sensitivity, anxiety, hostility, psychoticism, changed appetite, sleep disturbance, and suicidal ideation were lower and concentration better than in other types of recurrent depression. The results of the study indicate that it is important for health care services to follow up mothers who have had previous episodes of depression, including DDP and PPD, after delivery. If a mother has had DDP, she needs care already during pregnancy and usually also during the baby blues period. As it is possible that a mother will experience her first depression later, all mothers should be screened within six weeks of childbirth at the latest. Screening alone is not effective; it is also important to pay special attention to feelings of hopelessness and the diversity of symptoms with PPD. Furthermore, it is also important to give mothers information about PPD and to discuss the symptoms with them in order to raise their awareness of this disorder and possible new episodes in the future.

## Abbreviations

BDI: Beck depression inventory
BHS: Beck hopelessness scale
DDP: Depression during pregnancy
DSM-IV: Diagnostic and statistical manual of mental disorders, fourth edition
EPDS: Edinburg postnatal depression scale
MDE: Major depressive episode
PPD: Postpartum depression
SCID-1: Structured clinical interview
SCL-90: Symptoms checklist 90
SD: Standard deviation
OR: Odds ratio
CL: Confidence limits
SPSS: Statistical package for the social sciences
